# Altered Subcellular Localization of Heat Shock Protein 90 Is Associated with Impaired Expression of the Aryl Hydrocarbon Receptor Pathway in Dogs

**DOI:** 10.1371/journal.pone.0057973

**Published:** 2013-03-05

**Authors:** Frank G. van Steenbeek, Bart Spee, Louis C. Penning, Anne Kummeling, Ingrid H. M. van Gils, Guy C. M. Grinwis, Dik Van Leenen, Frank C. P. Holstege, Manon Vos-Loohuis, Jan Rothuizen, Peter A. J. Leegwater

**Affiliations:** 1 Department of Clinical Sciences of Companion Animals, Faculty of Veterinary Medicine, Utrecht University, Utrecht, The Netherlands; 2 Department of Pathobiology, Faculty of Veterinary Medicine, Utrecht University, Utrecht, The Netherlands; 3 Molecular Cancer Research, University Medical Centre Utrecht, Utrecht, The Netherlands; French National Centre for Scientific Research, France

## Abstract

The aryl hydrocarbon receptor (AHR) mediates biological responses to toxic chemicals. An unexpected role for AHR in vascularization was suggested when mice lacking AHR displayed impaired closure of the ductus venosus after birth, as did knockout mice for aryl hydrocarbon receptor interacting protein (AIP) and aryl hydrocarbon receptor nuclear translocator (ARNT). The resulting intrahepatic portosystemic shunts (IHPSS) are frequently diagnosed in specific dog breeds, such as the Irish wolfhound. We compared the expression of components of the AHR pathway in healthy Irish wolfhounds and dogs with IHPSS. To this end, we analyzed the mRNA expression in the liver of *AHR*,*AIP*, *ARNT*, and other genes involved in this pathway, namely, those for aryl hydrocarbon receptor nuclear translocator 2 (*ARNT2*), hypoxia inducible factor 1alpha (*HIF1A*), heat shock protein 90AA1 (*HSP90AA1*), cytochromes P450 (*CYP1A1, CYP1A2*, and *CYP1B1*), vascular endothelial growth factor A (*VEGFA*), nitric oxide synthesase 3 (*NOS3*), and endothelin (*EDN1*). The observed low expression of *AHR* mRNA in the Irish wolfhounds is in associated with a LINE-1 insertion in intron 2, for which these dogs were homozygous. Down regulation in Irish wolfhounds was observed for *AIP*, *ARNT2*, *CYP1A2*, *CYP1B1* and *HSP90AA1* expression, whereas the expression of *HIF1A* was increased. Immunohistochemistry revealed lower levels of AHR, HIF1A, and VEGFA protein in the nucleus and lower levels of ARNT and HSP90AA1 protein in the cytoplasm of the liver cells of Irish wolfhounds. The impaired expression of HSP90AA1 could trigger the observed differences in mRNA and protein levels and therefore explain the link between two very different functions of AHR: regulation of the closure of the ductus venosus and the response to toxins.

## Introduction

The ductus venosus is an embryonic vessel connecting the vena porta and vena cava and allows blood to flow from the placenta to the lungs and heart without traversing the liver sinusoids. The vessel closes within a few days after birth, thereby ensuring that the liver becomes fully functional [1]. Complete closure occurs within 6 to 9 days in healthy dogs [2]. However, in some purebred dogs, especially large and giant dogs such as Irish wolfhounds, the ductus venosus sometimes fails to close because of a genetic disorder [3]. A permanently patent ductus venosus, called an intrahepatic portosystemic shunt (IHPSS), leads to portosystemic bypass of venous hepatic perfusion, resulting in impaired growth and function of the liver and no clearance of intestinal metabolites, such as ammonia, from portal blood [4]. The prevalence of IHPSS in Irish wolfhounds is 2.1–3.4% [5], [6] and the disease has a polygenic mode of inheritance [7]. IHPSS is a rare disease in humans, with only 89 cases reported to date (listed in [3]).

Mouse knockout studies indicate that the aryl hydrocarbon receptor (AHR) and its downstream pathway might underlie this disease [8]. AHR displays tissue specific functions. In hepatocytes AHR signaling mediates adaptive and toxic responses to dioxins and other AHR agonists [9], [10]. In endothelial/hematopoietic cells AHR has been shown to be necessary for closure of the ductus venosus [8]. A patent ductus venosus was found in all knockout mice for AHR [8], [11] indicating a monogenic effect. In the absence of ligands, AHR forms a complex with aryl hydrocarbon receptor interacting protein (AIP), heat shock protein 90 kDa alpha (cytosolic), class A member 1 (HSP90AA1), and p23 proteins [9]. AIP is also involved in regulating closure of the ductus venosus. Comparable with the fully penetrant mutation in *AHR*, 83% of *AIP* (−/−) mice are affected by IHPSS [12].

Upon exposure to xenobiotics, AHR heterodimerizes with aryl hydrocarbon receptor nuclear translocator (ARNT) [13] and activates transcription of several cytochrome P-450 (CYP) subtype genes, of which *CYP1A1*, *CYP1A2,* and *CYP1B1* are considered most important. ARNT plays an essential role in developmental angiogenesis by dimerizing with HIF1A, which leads to the expression of proangiogenic factors such as vascular endothelial growth factor α (VEGFA), nitric oxide synthase 3 (NOS3), and endothelin 1 (EDN1) (Figure S1). Because of this role, *ARNT* null mice die during embryonic development [14]. Another factor that plays a central role is HSP90AA1, which forms a cytosolic complex with AHR in hepatocytes [15], and which is also essential for the regulation of *HIF1A* activation [16]. Closure of the ductus venosus in newborn lambs seems to be regulated by *EDN1* which functions as a potent constrictor of both sphincter and extrasphincter sections of the ductus. Prostaglandins cause a dilating affect and might also influence *EDN1* activity [17]. The physiologically comparable process of closure of the ductus arteriosus (a fetal shunt connecting the pulmonary artery with the aorta allowing blood to bypass the unexpanded lungs) appears to be mediated by *cytochrome P-450 3A13* and *EDN1* [18]. However, *CYP3A13* gene expression was found to be increased in mouse livers on day 20 after birth, indicating a late response in liver development [19].

The genes and pathways mentioned above are involved in IHPSS in mice and should be considered important candidates for the human and canine forms of the disease. The aim of this study was to investigate the AHR pathway at the DNA, mRNA, and protein level in dogs with IHPSS due to a persistent ductus venosus and in healthy control dogs.

## Materials and Methods

### Animals

All dogs were kept privately as companion animals. Written informed consent was obtained from all the owners of participants in our study. The dogs were presented to the Department of Clinical Sciences of Companion Animals, Utrecht University, either for population screening for the occurrence of portosystemic shunts [7] or as clinical cases with signs of liver dysfunction in which IHPSS was diagnosed. Blood samples were drawn from the jugular vein. Liver samples were collected from dogs during surgical attenuation of an IHPSS or extrahepatic portosystemic shunt (EHPSS) or at immediate post-mortem examination from healthy dogs (controls) from other, non liver-related, experiments. Wedge biopsies were snap frozen in liquid nitrogen or fixed in RNA later (Ambion, Inc., Austin, Texas) for RNA isolation; matching wedge biopsies were fixed in 10% neutral buffered formalin and embedded in paraffin for immunohistochemistry.

Genomic DNA was isolated from EDTA blood using the salt extraction method [20] or from formalin-fixed, paraffin-embedded tissue, using the DNA mini Kit (Qiagen, Venlo, the Netherlands). After isolation, DNA was frozen at −20°C until use. Liver tissue was lysed in 1 ml of TRIzol® reagent. Total RNA was isolated and chromosomal DNA was removed in accordance with the manufacturer’s instructions (Qiagen). RNA quality and quantity was determined on a nanochip (Bioanalyzer, Agilent Technologies, Santa Clara, US). cDNA was synthesized using the BioRad iScript Synthesis kit (BioRad, Veenendaal, the Netherlands). The procedures were approved by Utrecht University’s Ethical Committee, as required by Dutch legislation (ID 2007.III.08.110).

### Genomic DNA Sequence Analysis

DNA sequence analysis was performed on the exons including the splice sites of *AHR* in 33 dogs affected with IHPSS from 8 breeds and 51 controls from the same breeds. Additional DNA sequencing for *CYP1B1*, *HIF1A* and *HSP90AA1* was performed on 8 Irish wolfhounds affected by IHPSS, 8 healthy Irish wolfhounds, and 2 control dogs. Standard amplification was performed using PCR-mix containing 1× Platinum® PCR Buffer, dNTPs 0.5 µM each, 2 mM MgCl_2_, 0.5 mM of each forward and reverse primer, 1 Unit Platinum® Taq DNA polymerase (Invitrogen, Carlsbad, CA) and 50 ng of gDNA in a reaction volume of 25 µl. The thermal cycling protocol consisted of a 5-min denaturation step at 95°C, 35 cycles of 30 s at 95°C, 30 s at annealing temperature, 30 s at 72°C, and a final elongation step at 72°C for 10 min. For amplification of fragments exceeding 2000 bp, Phusion ™ Hot Start High-Fidelity DNA Polymerase (Thermo Scientific, Lafayette, US) was used. The reaction mix contained 1× Phusion™ GC Buffer, dNTPs 0.5 µM each, primer mix 0.5 mM each, 3% DMSO, 0.02 U/µl Phusion™ Hot start DNA polymerase, and 50 ng gDNA. Cycling conditions were 10 min at 98°C, 35 cycles of 30 s at 98°C, 30 s at 56°C, 7 min at 72°C, and a final elongation step at 72°C for 10 min. All amplifications were performed on an ABI 9700 Thermal Cycler (Applied Biosystems, Foster City, CA). Primers are listed in Table S1. DNA sequence reactions were performed using BigDye v3.1 according to the manufacturer’s (Applied Biosystems) instructions on an ABI3130XL and analyzed in Lasergene (version 9.1 DNASTAR). The obtained sequences were compared with DNA sequences in databases with BLASTn [21].

### Southern Blot

Genomic DNA was digested at 37°C for 4 h with *HindIII* and *PstI* restriction enzymes and was separated on 0.8% agarose TAE gel. The DNA was denatured by soaking the gel in 0.6 M NaCl/0.4 M NaOH for 1 h and then transferred overnight to hybond-N nylon membrane (Amersham, GE Healthcare, Diegem, Belgium) by capillary transfer method [22] using 0.4 M NaOH. The filter was dried by heating at 80°C for 2 h. The probe was obtained by PCR of exon 2 (fwd 5′-CAGCATTTTCTCAAGATGGG-3′ rev 5′-ATTGGAAGGAGAAGTGGAAC-3′) of *AHR* from a healthy control, which resulted in a 589-bp fragment that was purified with the QIAquick® PCR Purification Kit (Qiagen). The probe was radioactively labeled using the Megaprime DNA Labeling system (GE Healthcare) and 25 ng of denatured probe and [α-P32]dATP (Hartmann Analytics GmbH, Braunschweig, Germany). The labeled probe was purified on a Sephadex G-50 NICK Column (GE Healthcare). Hybridization was done overnight at 60°C. The blot was washed in a series of 5 steps from 2×SSPE/0.1% SDS to 0.1×SSPE/0.1% SDS for 20 min at 60°C.

### Linkage Analysis

To analyze the linkage between genes of interest and the shunt phenotype, we designed primers for genotyping of microsatellites in close chromosomal vicinity of *AHRR*, *ARNT*, *AIP*, *CYP1A1/CYP1A2*, *CYP1B1*, *EDN1*, *HIF1A*, *HSP90AA1*, *NOS3,* or *VEGFA,* respectively. The distance between *CYP1A1* and *CYP1A2* is less than 30 kb, hence two microsatellites were used to cover both genes at once. 500 kb of genomic DNA up- and downstream of the genes was downloaded from NCBI (CanFam2.1) and masked for simple repeats using repeat masker (www.repeatmasker.org). The masked sequences were analyzed with tandem repeat finder [23] to detect microsatellite repeats. Two microsatellites were selected for each gene and primers were designed with Perlprimer v1.1.13 (Table S1).

**Figure 1:**
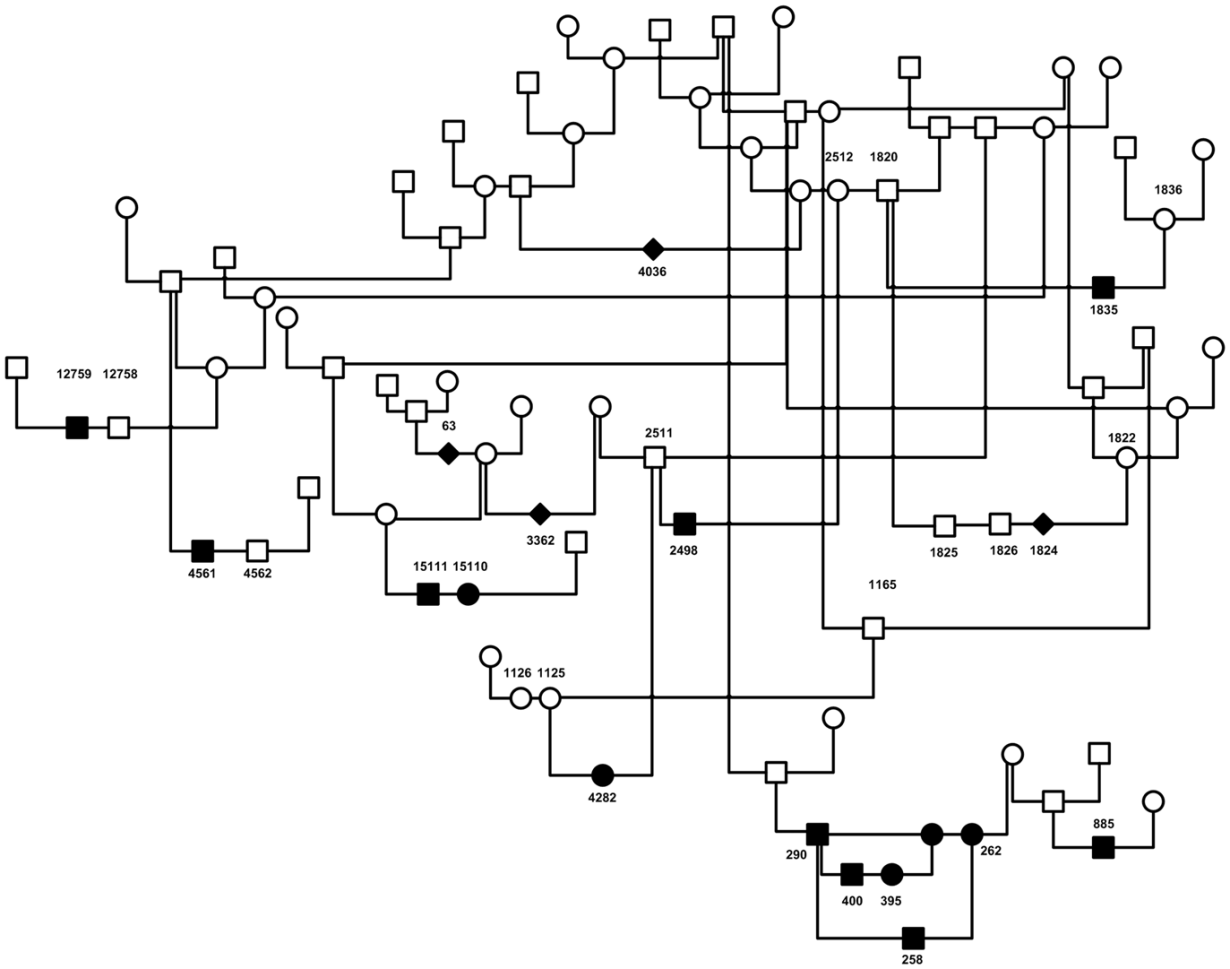
Pedigree of Irish Wolfhounds used for linkage analysis. Related Irish wolfhounds used to genotype 20 microsatellites. DNA was available from the numbered individuals. Filled symbols are affected dogs, open symbols represent healthy dogs.

Samples came from a pedigree containing 17 cases with and 13 healthy dogs without a shunt (Figure 1). A phage M13-based tag (GTTTTCCCAGTCACGAC) was added at the 5′ end of forward primers. PCR amplification was carried out in a reaction volume of 15 µl containing 25 ng of genomic DNA, 1 µM M13-tagged forward primer, 10 µM reverse primer, 10 µM M13-based tag primer labeled at the 5′ end with 6-FAM (Eurogentec, Maastricht, the Netherlands), 1× PCR gold buffer (Applied Biosystems), 2.5 mm MgCl_2_, 0.2 mM dNTPs and 0.3 U Amplitaq Gold. Thermal cycling was performed in a ABI 9700 (Applied Biosystems) with the following program: 5 min at 95°C, followed by 10 cycles of 30 s at 95°C, 15 s at the annealing temperature, 30 s at 72°C, then another 25 cycles of 30 s at 9 2°C, 15 s at the annealing temperature, and 30 s at 72°C. The program was completed with 10 min at 72°C. Genotypes were obtained on an ABI3130XL and scored using GeneMapper Software (version 4.0). Analysis was performed in Superlink [24] using the two-point two-loci option. Disease gene frequencies were set at 0.05 and the mode of inheritance at 0.01 for two risk alleles or less and 0.99 for three risk alleles or more.

### mRNA Quantification

#### qPCR

To measure RNA expression in liver tissue, primers were designed for the genes for AHR, ARNT, ARNT2, AIP, HIFIA, HSP90AA1, cytochrome P450 (CYP1A1, CYP1A2, and CYP1B1), VEGFA, NOS3, and EDN1. Perlprimer v1.1.14 was used for primer design on Ensembl annotated transcripts and the amplicon was tested for secondary structures using MFold [25]. Gradient PCRs were performed to determine the optimum temperature for obtaining 100% efficiency. Primer specificity was validated *in silico* (BLAST specificity analysis) and empirically (DNA sequencing, gel electrophoresis and melting profiles). qPCR reactions were performed in 25-µl duplicates containing 0.5×SYBR Green-Supermix (BioRad), 0.4 µM primer, and 1 µl cDNA. For normalization, five reference genes were used based on their stable expression in the liver, namely, genes for hypoxanthine phosphoribosyl transferase (*HPRT*), beta-2-microglobulin (*B2M*), heterogeneous nuclear ribonucleoprotein H1 (*hnRPH*), beta-glucuronidase (*GUSB*), and ribosomal proteins S5 (*RPS5*) [26]. GeneNorm [27] was used to establish stability. Primers for reference genes and genes of interest including their optimum temperature are listed in Table S2. Cycling conditions were a 3-min Taq polymerase activation step on 95°C, followed by 45 cycles of 10 s at 95°C to denature, and 30 s at T_m_ for annealing and elongation. For some products a 3-step protocol was used including 3-min Taq polymerase activation step at 95°C, followed by 45 cycles of 10 s at 95°C to denature, 30 s at T_m_ for annealing, and 30 s at 72°C for elongation. All experiments were conducted on a MyiQ Single-Colour Real-Time PCR Detection System (BioRad). A 4-fold standard dilution series of a pool containing all samples used for analysis was used to determine relative expression. cDNA originating from liver tissue of 8 healthy beagles, 8 dogs of various breeds with IHPSS (Table S3), and 8 Irish wolfhounds with IHPSS were used for expression analysis. Negative controls remained negative [28]. Data analysis was performed in IQ5 Real-Time PCR detection system software (BioRad). Gene expression was normalized by using the average relative amount of the reference genes. Log values of normalized relative gene expression were used to obtain normal distribution. A Wilcoxon rank sum test was performed in R statistics package 2.14.0 (http://www.R-project.org) to determine significance of differential expression.

#### Microarray expression profiling

Liver tissue from 2 healthy dogs, 32 dogs with EHPSS, and 15 dogs with IHPSS (Table S3) were used for total RNA isolation using an RNeasy Mini Kit (Qiagen). DNase treatment was performed using an on-column DNase digestion. RNA quality and quantity was determined on a nanochip (Bioanalyzer, Agilent Technologies). Samples with a RIN value above 8.0 were used. A common reference pool was constructed by pooling RNA isolated from healthy liver. Agilent Canine Gene Expression Microarray V1 containing 42,034 60-mer probes in a 4×44K layout was used to determine genome wide expression on 3 µg of total RNA of each sample hybridized to the common reference. RNA amplification and labeling were performed on an automated system (Caliper Life Sciences NV/SA, Belgium) [29]. Dye swap of Cy3 and Cy5 was performed to reduce dye bias. Hybridizations were done on a HS4800PRO system supplemented with QuadChambers (Tecan Benelux B.V.B.A. Mechelen, Belgium) using 1 µg labeled cRNA per channel [30].

Hybridized slides were scanned on an Agilent scanner (G2565BA) at 100% laser power, 30% PMT and with automated data extraction, using Imagene 8.0 (BioDiscovery). Normalization was performed with Loess on mean spot intensities [31], and dye bias was corrected based on a within-set estimate [32]. Data were analyzed using ANOVA (R version 2.2.1/MAANOVA version 0.98–7) [33]. Correction for multiple testing (Permutation F2-test using 5,000 permutations) was performed.

### Immunohistochemical Analysis for Localisation and Quantification of Proteins

Immunohistochemistry (IHC) was performed for AHR, ARNT, HIF1A, CYP1A1, CYP1A2, CYP1B1, VEGFA, NOS3, and HSP90AA1 on liver samples of healthy beagles (n = 6), Irish wolfhounds (n = 11, 2 with IHPSS, 9 healthy), and arbitrary selected dogs of other breeds with an IHPSS (n = 6), and dogs with an EHPSS (n = 6). Samples from dogs with IHPSS and dogs with an EHPSS were compared to identify specific effects related to the secondary effects of blood bypassing the liver tissue on protein expression. Antibody characteristics, manufacturers, dilutions, and protocol specifications are given in Table S4. Five-micrometer sections of paraffin-embedded liver tissue were deparaffinized in xylene and rehydrated in an ethanol to water series. Heat-induced antigen retrieval was performed with 10 mM citrate buffer (pH 6.0) or 10 mM Tris with 1 mM EDTA (pH 8.0) at 98°C in a water bath, followed by cooling at room temperature (RT) for 30 min (Table S4). Antigen retrieval by enzymatic digestion was performed with proteinase K (Dakocytomation, Glostrup, Denmark) for 10 min at RT. Dual endogenous enzyme block (Dakocytomation) was used (10 min, RT) to quench endogenous peroxidase activity, and background staining was blocked with 10% normal goat serum (Sigma-Aldrich, St. Louis, US) (30 min). Sections were incubated with the labeled secondary antibody Envision (Dakocytomation) for 1 h at RT. The signal was developed in 0.06% 3,3′-diaminobenzidine (DAB) solution (Dakocytomation) for the indicated time (Table S4) and counterstained with hematoxylin QS (Vector Laboratories, Burlingame, CA). Replacement of primary antibody with washing buffer served as negative control. All tissues were stained in batch per antibody to avoid technique-induced differences.

All immunohistochemically stained sections were evaluated by one board-certified pathologist (GCMG) who was unaware of the origin of the samples, using a semi-quantitative scoring system based on the intensity and localization of staining, with grading as follows: 0, absent; 1, mild positive staining; 2, moderate positive staining; 3, strong positive staining. If different histological elements (hepatocytes, bile ducts, Kupffer cells) were stained, then staining in these elements was scored separately. Information on acinar localization (zone 1, 2, or 3) was also collected. The average intensity score of each group (i.e., Irish wolfhounds, dogs with an IHPSS, dogs with an EHPSS, and control dogs) was calculated.

All data were analyzed using R statistics package 2.14.0. Overall differences in intensity scores between Irish wolfhounds and remaining samples including dogs with an IHPSS, dogs with an EHPSS, and control dogs were tested with the Wilcoxon rank sum test, with P<0.05 being considered statistically significant. Differences in protein localization were identified by detecting the pattern of protein expression in each group.

## Results

### Rearrangement of AHR

**Figure 2:**
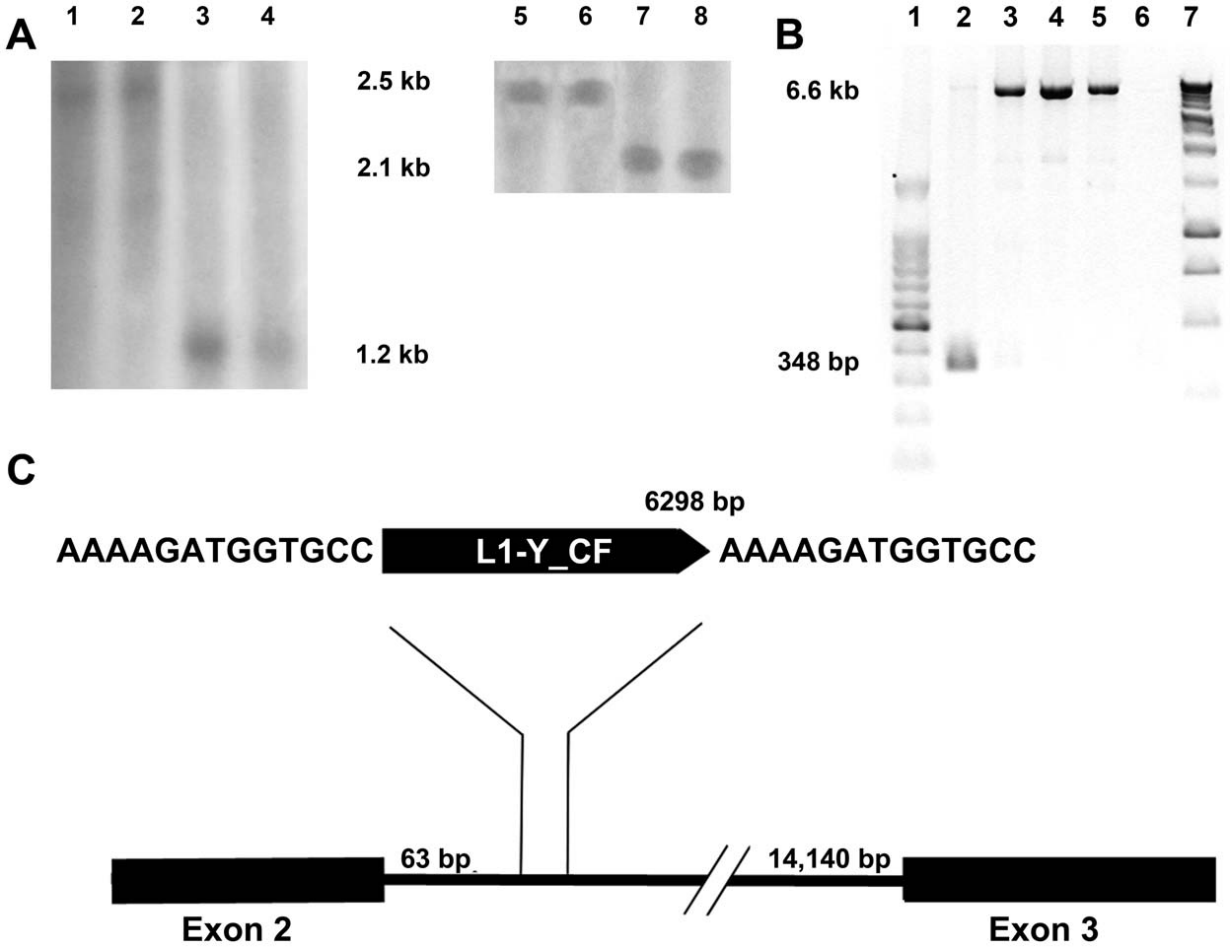
LINE-1 insertion in intron 2 of *AHR* in Irish Wolfhounds. Southern blot analysis (A) with HindIII (lanes 1–4) and PstI (lanes 5–8) digested genomic DNA. Digestion with HindIII resulted in an increase in size of about 2000 bp in Irish wolfhounds (lanes 1 and 2) compared to healthy beagles (lanes 3 and 4). Using PstI as restriction enzyme results in a small increase in size of approximately 400 bp in Irish wolfhounds (lanes 5 and 6) compared to healthy beagles (lanes 7 and 8). The observed size differences are smaller than the actual rearrangement due to the presence of restriction enzyme sites in the rearranged DNA fragment. Gel electrophoresis of PCR products (B) with forward primer in exon 2 and reverse primer downstream intron 2. The use of Phusion ™ Hot Start High-Fidelity DNA Polymerase in combination with GC-buffer was required to amplify this fragment. An increase in intron size was detected in all used gDNA samples of Irish wolfhounds. Lane 1∶1 kb DNA ladder (Promega, Leiden, the Netherlands); 2: normal control; 3–5: genomic DNA of Irish wolfhounds; 6: negative control; 7∶100 bp DNA ladder (Promega). A 6298 bp long LINE-1 insertion named L1-Y_CF (C) is located 63 bp downstream of exon2. It contains a 13 bp long duplication site specific for LINE-1 insertions. In both open reading frames of this retrotransposon deletions were detected causing frame shifts and premature stop codons. Restriction sites were found for HindIII at 1908 bp and for PstI at 447 bp.

As a first step to investigate the role of AHR in IHPSS we analyzed the exons of the gene in affected dogs. DNA sequence analysis of *AHR* did not reveal differences between the gene of the canine reference genome and that of dogs with an IHPSS. However, amplification of the fragment containing exon 2 in all affected and healthy Irish wolfhounds failed, indicating a rearrangement in this region. Southern blot analysis (Figure 2A) and a PCR adapted for long DNA fragments indicated that intron 2 was larger by more than 6 kb in Irish wolfhounds (Figure 2B). DNA sequence analysis of the PCR product revealed an insertion of 6298 bp, positioned only 63 bp downstream of exon 2 (Figure 2C). BLASTn comparison of the insert against a database of repeats in mammalian DNA showed high similarity to L1-Y_CF. The LINE-1 insertion included a target site duplication of 13 bp, a 5′ UTR, an open reading frame (ORF1) coding for a high-affinity RNA-binding protein, an ORF2 containing both endonuclease and reverse transcriptase activities, and a 3′ UTR and a poly(A) tail. In both ORFs, a frame shift caused by a single nucleotide deletion was detected, resulting in a premature stop codon. The insert was found homozygously in all but one of the Irish wolfhounds. Only one healthy dog was heterozygous for the rearrangement. DNA sequencing of *CYP1B1, HIF1A,* and *HSP90AA1* did not reveal differences between healthy dogs and dogs with an IHPSS that could have caused the disorder.

### Linkage Analysis

On the basis of test matings of Irish wolfhounds with an IHPSS, we previously proposed a digenic, triallelic mode of inheritance, thus two interacting loci in which a total of at least three risk alleles should be present [7]. In order to determine whether IHPSS could be explained by such an interaction between genes of the AHR pathway, we genotyped dogs from an IHPSS pedigree with 16 polymorphic microsatellite markers situated close to genes of the AHR pathway (Figure 1). The maximally obtainable LOD score with the available samples was 1.8. This LOD score was determined by assuming genotypes of the available samples in two loci according to the postulated model. Using the two-point, two-loci option in Superlink [24], we calculated LOD scores varying between 0 and 1.7 (Table S5). The highest scores were obtained with combinations of markers for HSP90AA1 and the genes *CYP1A1 and CYP1A2,* which are situated close to each other.

### mRNA Quantification

**Figure 3:**
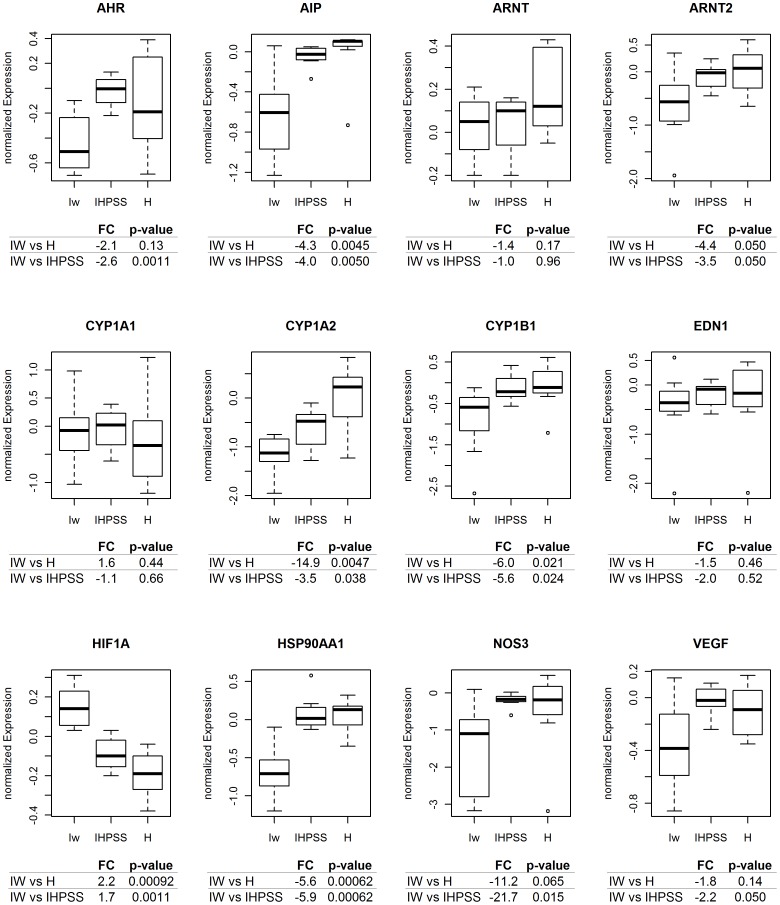
Comparison of *AHR* pathway gene expression in livers with a shunt and normal livers. The pattern of expression of selected genes in liver samples from Irish wolfhounds with an intrahepatic shunt (Iw), dogs with intrahepatic portosystemic shunts (IHPSS), and healthy dogs (H). The thick black line represents the median (50th percentile); the first and third quartile (25th and 75th percentile, respectively) are displayed. Outliers are depicted with an open dot, representing values higher than 1.5 times the interquartile range. HIF1A was found to be significantly upregulated in Irish wolfhounds with IHPSS, whereas *AHR*, *AIP*, *ARNT2, CYP1A2, CYP1B1, HSP90AA1, NOS3,* and *VEGFA* were significantly down regulated. No differences were observed in *ARNT*, *CYP1A1*, and *EDN1*. FC = fold change.

To study the expression of the genes of the AHR pathway, the mRNA levels of 12 genes were determined by qPCR (Figure 3). *ARNT*, *CYP1A1,* and *EDN1* expression was similar in dogs with an IHPSS and control dogs. In contrast, *HIF1A* was upregulated (2.2-fold change ), whereas *AHR*, *AIP*, *ARNT2*, *CYP1A2*, *CYP1B1*, *HSP90AA1*, *NOS3,* and *VEGFA* were down-regulated (1.8 to 14.9-fold change) in Irish wolfhounds with IHPSS. In microarray expression profiling the 12 target genes were compared between IHPSS and controls and between EHPSS and controls (Table S6). In dogs with EHPSS a developmental vascular anomaly is formed by which the extrahepatic portal system is connected with the caudal vena cava or (hemi)azygos vein. For both shunts the functional consequences, virtual absence of portal perfusion of the liver parenchyma, and clinical signs are similar. Functional consequences include hypoplasia of the liver and elevated ammonia and bile acid levels in the systemic blood of the cases compared to healthy individuals. To avoid measuring mRNA expression differences due to unspecific effects caused by absence of portal perfusion *per se*, samples of dogs with EHPSS were included [3]. Microarray data have been deposited in NCBI’s Gene Expression Omnibus [34] and are accessible through GEO Series accession number GSE39005 (http://www.ncbi.nlm.nih.gov/geo/query/acc.cgi?acc=GSE39005). As *CYP1A1* and *CYP1A2* were down-regulated in dogs with IHPSS or EHPSS in the microarray analysis, this down-regulation is probably an effect secondary to toxification caused by the lack of hepatic clearance of ammonia and bile acids. CYP1B1, NOS3 and VEGFA are downstream products of the investigated pathway. Down regulation of these products might be effects of impaired regulation of upstream genes. The lower expression of *HSP90AA1* is remarkable, because of its role in the classical AHR pathway and the HIF1A pathway.

### Immunohistochemistry

**Figure 4:**
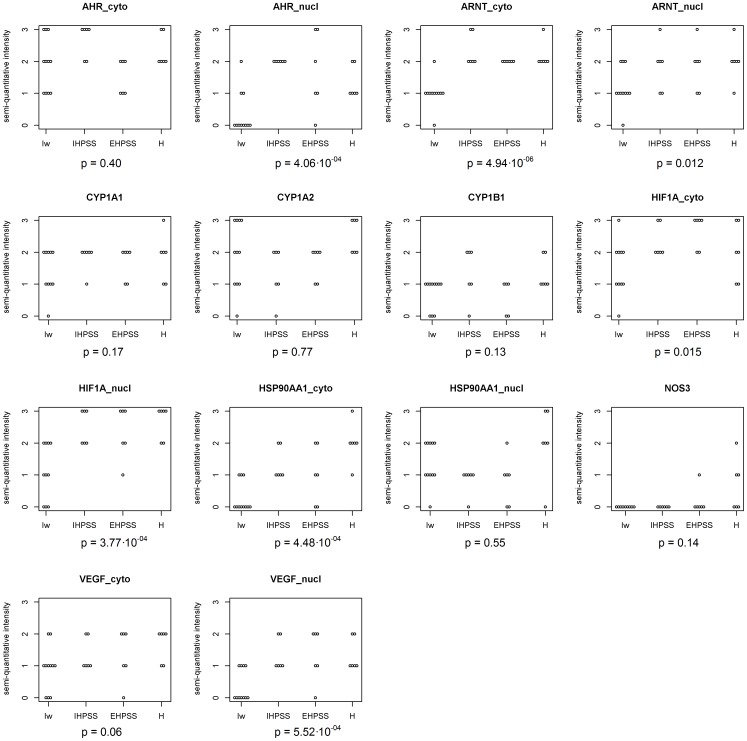
Immunohistochemistry of AHR pathway proteins in liver samples from healthy dogs, dogs with extrahepatic (EPHSS) or intrahepatic (IPHSS) portosystemic shunts, and Irish wolfhounds. The semi-quantative scoring in hepatocytes from Irish wolfhounds (Iw, n = 11), dogs with intrahepatic portosystemic shunts (IHPSS, n = 6), dogs with extrahepatic portosystemic shunts (EHPSS, n = 11) and healthy beagles (H, n = 6). The indicated p-values are obtained comparing Irish wolfhounds with the other groups as a whole.

**Figure 5:**
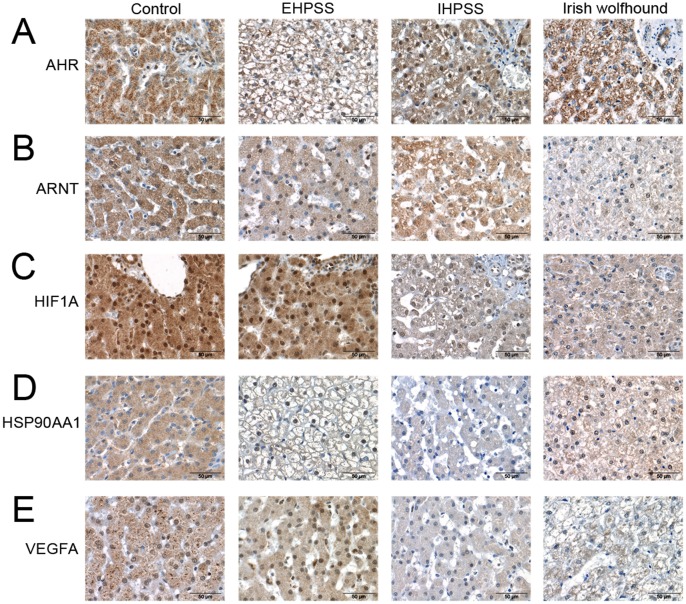
Significant immunohistochemical staining of liver tissue. Representative images of immunoreactivity in sections of formalin-fixed, paraffin-embedded liver samples from control dogs, dogs with extrahepatic and intrahepatic portosystemic shunts, and Irish Wolfhounds. Immunoreactivity against the aryl hydrocarbon receptor (AHR)(A), aryl hydrocarbon receptor nuclear translocator (ARNT)(B), hypoxia inducible factor 1alpha (HIF1A)(C), heat shock protein 90kDa alpha (cytosolic), class A member 1 (HSP90AA1)(D), and vascular endothelial growth factor A (VEGF)(E) is visible in variable intensity in the cytoplasm and/or nuclei of hepatocytes.

Immunohistochemistry was performed to investigate protein expression, measured semi-quantitatively, and protein localization. The intensity of hepatocyte staining for AHR, ARNT, HIF1A, HSP90AA1, and VEGFA was different in Irish wolfhounds and control dogs (Figure 4), whereas there were no differences in the intensity of staining for the downstream products CYP1A1, CYP1A2, CYP1B1, and NOS3. These five differently staining proteins were down regulated in the Irish wolfhounds compared to both healthy controls and dogs with EHPSS indicating that the differences were not caused by secondary effects of shunting. Staining for AHR was lower in the nuclei of hepatocytes of Irish wolfhounds than in control dogs (Figure 5A), but was significantly stronger in the nuclei of hepatocytes from dogs with an IHPSS or EHPSS and in hepatocytes from healthy control dogs (p = 8.0×10^−5^). Hepatocyte nuclei and cytoplasm stained positively for ARNT (Figure 5B), but staining was less intense in Irish Wolfhounds than in the other groups of dogs (p = 4.9×10^−8^). HIF1A staining was present in nearly all hepatocyte nuclei, but nuclear staining was less intense in Irish wolfhounds than in control dogs (p = 3.9×10^−5^) (Figure 5C). Healthy hepatocytes displayed weak staining for HSP90AA1 (Figure 5D), but staining was distinctly less intense in the cytoplasm of hepatocytes from Irish wolfhounds (p = 1.2×10^−4^). Staining for VEGFA in the nuclei was less intense in hepatocytes from Irish wolfhounds than in hepatocytes from dogs with an EHPSS or IHPSS and healthy controls (p = 1.2×10^−4^) (Figure 5E). All p-values indicated here were based on the comparison of the Irish wolfhounds against the samples from other breeds. The decreased expression of AHR and HIF1A in the nuclei indicates a problem in trafficking these proteins. This clearly fits the impaired expression of HSP90AA1 which plays an important role in this translocation. Also the nuclear and cytoplasmic decrease of ARNT which cooperates with HSP90AA1 could explain decreased expression of VEGFA.

## Discussion

Given its role in closure of the ductus venosus, we hypothesized that AHR and its downstream pathway are involved in IHPSS in dogs. In the present study, liver tissue from affected and healthy control dogs was used for microarray expression profiling, qPCR, and immunohistochemical analysis of the genes involved in this pathway. Dogs with a portosystemic shunt have poor growth and function of the liver after birth, and changes secondary to portosystemic shunting are expected to alter the expression of genes and proteins in the liver. This would complicate analysis of whether abnormalities are the cause (associated with the causal mutation of the genetic disease) or effect (due to portal hypoperfusion) of the disease. Therefore, liver tissues of dogs affected with EHPSS were used as a control to correct for secondary effects which should be the same as in IHPSS. The genetic background, however, the two conditions affect different purebred dog populations [3], are differently transmitted in affected families and are therefor genotypically different entities.

While the sequence of the *AHR* gene was identical in Irish wolfhounds with and without an IHPSS and in affected and healthy dogs of other breeds, Irish wolfhounds had a canine specific LINE1-insert named L1-Y_CF only 63 bp downstream of intron 2. In another study, a sense L1 insert of 4760 bp was found to strongly attenuate target gene expression [35]. The L1-Y_CF insert was 6298 bp, and a decreased expression of *AHR* was anticipated. Although the LINE1 seems to be inactivated due to a frame shift in both *ORF1* and *ORF2*, the level of expression of *AHR* in Irish wolfhounds was lower than that in healthy control dogs of other breeds. The location of the insert near the splice donor site and/or the altered structure of the intron may cause this decreased expression. As the insert was present in both healthy and affected Irish wolfhounds, it cannot be the single cause of the disease, but it might be a predisposing factor that increases the risk of the entire breed.

A simple Mendelian inheritance pattern has been ruled out for patency of the ductus venosus in Irish wolfhounds [7]. Instead, we postulated a model in which two loci interact to determine the phenotype. At least three risk alleles would need to be present in the two loci to cause IHPSS. To determine whether genes of the AHR pathway are involved, we analyzed closely situated polymorphic microsatellite markers to calculate the LOD score for linkage according to the postulated model. The highest LOD scores (1.7) were obtained for the region of the *CYP1A1/CYP1A2* gene pair and for *HSP90AA1;* these scores were close to the maximally obtainable score of 1.8. A cytochrome P-450 system has been postulated to be involved in the closure of the ductus venosus in lambs by virtue of its contractile effect in the sphincter region [36]. Which cytochrome P-450 subtype is responsible for this effect is still unknown, but *CYP1A1* and *CYP1A2* were recently found not to be responsible for the process in mice [37]. At this stage, we cannot conclude whether the *CYP1A1/CYP1A2* and *HSP90AA1* loci are responsible for the shunt phenotype because the linkage score was not conclusive. A larger set of dogs from pedigrees with IHPSS needs to be investigated to confirm the involvement of these loci.

The decrease in *AHR* mRNA expression and the lack of nuclear translocation were linked to a decreased expression of the downstream targets CYP1A2 and CYP1B1. *CYP1A2* expression was down regulated in dogs with an IHPSS, and Irish wolfhounds with an IHPSS had lower levels of CYP1A2 expression than other large-breed dogs with an IHPSS. This may be explained by the breed-specific LINE-1 insert causing down-regulation of *AHR* in Irish wolfhounds. Dogs of this breed have been reported to be prone to complications of thiobarbiturate anesthesia, because the drug is not metabolized, possibly because of hypofunction of hepatic cytochrome P450 [38]. The decreased expression of *CYP1A2* found in this study supports this supposition. Given the specific expression patterns of all P450s [19], it seems sensible to measure a broad spectrum of cytochromes to determine the cause of this metabolic defect.

Mice with a hypomorphic *ARNT* allele have the same phenotypic alterations as *AHR* knock-out mice [39]. However, hepatocyte-specific deletion of *ARNT* did not result in shunting, so hepatocyte *ARNT* would appear not to be related to AHR-mediated hepatovascular development. ARNT2, a homolog of ARNT mainly expressed in neurons, has feedback regulation of its activity. It forms functional complexes with *HIF1A,* restoring hypoxia-induced gene expression in *ARNT*-deficient hepatocytes [40], [41]. In contrast, ARNT2 appears not to be able to compensate for the loss of ARNT with regard to the response to xenobiotics [42]. While Irish wolfhounds with a LINE-1 insert did not show an altered *ARNT* expression, *ARNT2* expression was decreased. Thus the *ARNT2* feedback mechanism might be impaired in Irish wolfhounds, possibly affecting closure of the ductus venosus.

*HIF1A* was the only gene to be upregulated in Wolfhounds. While gene expression was upregulated 2.2-fold, HIF1A protein expression was decreased in the cytoplasm and nuclei of hepatocytes from Wolfhounds. A similar discordance between gene and protein expression was found for AHR. This is most likely caused by the decreased amount of HSP90AA1. The down-regulation at the mRNA and protein level of HSP90AA1 is expected to affect both toxicological [15] and vascularization [16] processes. Binding of HSP90AA1 with AHR is essential for nuclear translocation [43], whereas binding of the HSP90 heterocomplex with HIF1A prevents the non-specific degradation of this highly unstable protein [44]. No coding variations were found in HSP90AA1. Whether epigenetic modification influences closure of the ductus venosus remains to be elucidated, but HSP90AA1 might be the missing link connecting toxicological responses via AHR with the regulation of vascularization.

Endothelin was postulated to have a role in the closure of the ductus venosus and the ductus arteriosus, but we found no evidence to support this in our material. However, it should be noted that all our liver samples were obtained from dogs that were several months old. Ideally, liver tissue should be collected within days of birth to investigate the biological initiators of closure of the ductus venosus. One study reported that the ductus was virtually closed in most Irish wolfhound pups on day 6 after birth, although the ductus was still partially present in 23% of pups. Closure was complete in all pups by day 9 [2]. Based on our findings, we cannot conclude that AHR and its downstream pathway are directly involved in the abnormal closure of the ductus venosus, but the decreased expression of AHR, and the lack of nuclear expression of the protein, in Irish wolfhounds might have a contributory role in delayed closure of the ductus venosus. To date, the impaired physiological process of ductus venosus closure, resulting in portosystemic shunting, has been investigated in experimental mice. The dog provides the opportunity to further study the genes regulating this process in a natural model, which may be relevant to unravel this rare disease in man.

## Supporting Information

Figure S1 **Overview of the combined Aryl hydrocarbon Receptor and Hypoxia Inducible Factor 1, alpha pathways.** The relationship between the AHR pathway and the HIF1A pathway. Both pathways share ARNT and HSP90AA1 as key regulators. HRE = hypoxia response element; XRE = xenobiotic response element.
(TIF)

Table S1 **primer sequences and annealing temperatures genomic DNA PCR.**
(DOCX)

Table S2 **primer sequences and annealing temperatures used for quantitative PCR.** *B2M* = β-2-Microglobulin, *GAPDH* = Glyceraldehyde-3-phosphatedehydrogenase, *GUSB* = beta-glucuronidase precursor, *HNRPH* = Heterogeneous nuclear ribonucleoprotein H, *HPRT* = hypoxanthine-guanine phosphoribosyltransferase, *RPS5* = Ribosomal protein S5, *AHR* = aryl hydrocarbon receptor, *AIP* = aryl hydrocarbon receptor interacting protein, *ARNT* = aryl hydrocarbon receptor nuclear translocator, *CYP1A1* = cytochrome P450, family 1, subfamily A, polypeptide 1, *CYP1A2* = cytochrome P450, family 1, subfamily A, polypeptide 2, *CYP1B1* = cytochrome P450, family 1, subfamily B, polypeptide 1, *EDN1* = Endothelin-1, *HIF1A* = Hypoxia-inducible factor 1 alpha, *HSP90AA1* = heat shock protein 90kDa alpha (cytosolic), class A member 1, *NOS3* = nitric oxide synthase, endothelial, *VEGFA* = Vascular endothelial growth factor α
(DOCX)

Table S3 **Samples from dogs with extrahepatic portosystemic shunts (EHPSS) or intrahepatic portosystemic shunts (IHPSS) used for microarray and qPCR.**
(DOCX)

Table S4 **Antibodies used for immunohistochemistry.** TE = Tris-Ethylenediaminetetraacetic acid, RT = room temperature, O/N = over night, PBS = Phosphate buffered saline, TBS = Tris buffered saline.
(DOCX)

Table S5 **LOD scores for linkage of shunt phenotype with candidate genes from the AHR pathway in a digenic model.** Genotypes of polymorphic microsatellites located close to candidate genes were analyzed assuming no recombinations occurred between markers and genes.
(DOCX)

Table S6 **Comparison of mRNA expression resulting from the microarray of genes of the AHR pathway.** Relative mRNA expression of genes of the AHR pathway detected in microarray. Iw = Irish wolfhound, IHPSS = intrahepatic portosystemic shunt, EHPSS = extrahepatic portosystemic shunt.
(DOCX)

## References

[1] Lohse CL, Suter PF (1977) Functional closure of the ductus venosus during early postnatal life in the dog. Am J Vet Res 38: 839–44. 560155

[2] Lamb CR, Burton CA (2004) Doppler ultrasonographic assessment of closure of the ductus venosus in neonatal irish wolfhounds. Vet Rec 155: 699–701. 15605536 10.1136/vr.155.22.699

[3] van Steenbeek FG, van den Bossche L, Leegwater PA, Rothuizen J (2012) Inherited liver shunts in dogs elucidate pathways regulating embryonic development and clinical disorders of the portal vein. Mammalian Genome: Official Journal of the International Mammalian Genome Society 23 76–84: 10.1007/s00335–011-9364-0. 10.1007/s00335-011-9364-0 PMC3275728 22052005

[4] van den Ingh TS, Rothuizen J, Meyer HP (1995) Circulatory disorders of the liver in dogs and cats. Vet Q 17: 70–6. 7571284 10.1080/01652176.1995.9694536

[5] Meyer HP, Rothuizen J, Ubbink GJ, van den Ingh TS (1995) Increasing incidence of hereditary intrahepatic portosystemic shunts in irish wolfhounds in the netherlands (1984 to 1992). Vet Rec 136: 13–6. 7900255 10.1136/vr.136.1.13

[6] Kerr MG, van Doorn T (1999) Mass screening of irish wolfhound puppies for portosystemic shunts by the dynamic bile acid test. Vet Rec 144: 693–6. 10420483 10.1136/vr.144.25.693

[7] van Steenbeek FG, Leegwater PA, van Sluijs FJ, Heuven HC, Rothuizen J (2009) Evidence of inheritance of intrahepatic portosystemic shunts in irish wolfhounds. J Vet Intern Med 23: 950–2. 19496918 10.1111/j.1939-1676.2009.0319.x

[8] Lahvis GP, Lindell SL, Thomas RS, McCuskey RS, Murphy C, et al (2000) Portosystemic shunting and persistent fetal vascular structures in aryl hydrocarbon receptor-deficient mice. Proc Natl Acad Sci U S A 97: 10442–7. 10973493 10.1073/pnas.190256997 PMC27043

[9] Mimura J, Fujii-Kuriyama Y (2003) Functional role of AhR in the expression of toxic effects by TCDD. Biochim Biophys Acta 1619: 263–8. 12573486 10.1016/s0304-4165(02)00485-3

[10] Walisser JA, Glover E, Pande K, Liss AL, Bradfield CA (2005) Aryl hydrocarbon receptor-dependent liver development and hepatotoxicity are mediated by different cell types. Proc Natl Acad Sci U S A 102: 17858–63. 16301529 10.1073/pnas.0504757102 PMC1308889

[11] Lahvis GP, Pyzalski RW, Glover E, Pitot HC, McElwee MK, et al (2005) The aryl hydrocarbon receptor is required for developmental closure of the ductus venosus in the neonatal mouse. Mol Pharmacol 67: 714–20. 15590894 10.1124/mol.104.008888

[12] Lin BC, Nguyen LP, Walisser JA, Bradfield CA (2008) A hypomorphic allele of aryl hydrocarbon receptor-associated protein-9 produces a phenocopy of the AHR-null mouse. Molecular Pharmacology 74 1367–1371: 10.1124/mol.108.047068. 10.1124/mol.108.047068 PMC2909677 18669605

[13] Fujii-Kuriyama Y, Mimura J (2005) Molecular mechanisms of AhR functions in the regulation of cytochrome P450 genes. Biochemical and Biophysical Research Communications 338 311–317: 10.1016/j.bbrc.2005.08.162. 10.1016/j.bbrc.2005.08.162 16153594

[14] Maltepe E, Schmidt JV, Baunoch D, Bradfield CA, Simon MC (1997) Abnormal angiogenesis and responses to glucose and oxygen deprivation in mice lacking the protein ARNT. Nature 386: 403–7. 9121557 10.1038/386403a0

[15] Perdew GH (1988) Association of the ah receptor with the 90-kDa heat shock protein. The Journal of Biological Chemistry 263: 13802–13805. 2843537

[16] Minet E, Mottet D, Michel G, Roland I, Raes M, et al (1999) Hypoxia-induced activation of HIF-1: Role of HIF-1alpha-Hsp90 interaction. FEBS Letters 460: 251–256. 10544245 10.1016/s0014-5793(99)01359-9

[17] Adeagbo AS, Kelsey L, Coceani F (2004) Endothelin-induced constriction of the ductus venosus in fetal sheep: Developmental aspects and possible interaction with vasodilatory prostaglandin. British Journal of Pharmacology 142 727–736: 10.1038/sj.bjp.0705849. 10.1038/sj.bjp.0705849 PMC1575056 15172962

[18] Baragatti B, Ciofini E, Scebba F, Angeloni D, Sodini D, et al (2011) Cytochrome P-450 3A13 and endothelin jointly mediate ductus arteriosus constriction to oxygen in mice. American Journal of Physiology.Heart and Circulatory Physiology 300 H892–901: 10.1152/ajpheart.00907.2010. 10.1152/ajpheart.00907.2010 21193583

[19] Peng L, Yoo B, Gunewardena SS, Lu H, Klaassen CD, et al (2012) RNA sequencing reveals dynamic changes of mRNA abundance of cytochromes P450 and their alternative transcripts during mouse liver development. Drug Metabolism and Disposition: The Biological Fate of Chemicals 40 1198–1209: 10.1124/dmd.112.045088. 10.1124/dmd.112.045088 PMC3362789 22434873

[20] Miller SA, Dykes DD, Polesky HF (1988) A simple salting out procedure for extracting DNA from human nucleated cells. Nucleic Acids Research 16: 1215. 3344216 10.1093/nar/16.3.1215 PMC334765

[21] Jurka J, Klonowski P (1996) Integration of retroposable elements in mammals: Selection of target sites. J Mol Evol 43: 685–9. 8995066 10.1007/BF02202117

[22] Maniatis T, Fritsch EF, Sambrook J (1982) Molecular cloning: A laboratory manual. United States of America: Cold Spring Harbor Laboratory.

[23] Benson G (1999) Tandem repeats finder: A program to analyze DNA sequences. Nucleic Acids Research 27: 573–580. 9862982 10.1093/nar/27.2.573 PMC148217

[24] Fishelson M, Geiger D (2002) Exact genetic linkage computations for general pedigrees. Bioinformatics (Oxford, England) 18 Suppl 1S189–98. 10.1093/bioinformatics/18.suppl_1.s189 12169547

[25] Zuker M (2003) Mfold web server for nucleic acid folding and hybridization prediction. Nucleic Acids Research 31: 3406–3415. 12824337 10.1093/nar/gkg595 PMC169194

[26] Brinkhof B, Spee B, Rothuizen J, Penning LC (2006) Development and evaluation of canine reference genes for accurate quantification of gene expression. Analytical Biochemistry 356 36–43: 10.1016/j.ab.2006.06.001. 10.1016/j.ab.2006.06.001 16844072

[27] Vandesompele J, De Preter K, Pattyn F, Poppe B, Van Roy N, et al (2002) Accurate normalization of real-time quantitative RT-PCR data by geometric averaging of multiple internal control genes. Genome Biology 3: RESEARCH0034. 12184808 10.1186/gb-2002-3-7-research0034 PMC126239

[28] Bustin S, Penning LC (2012) Improving the analysis of quantitative PCR data in veterinary research. Veterinary Journal (London, England : 1997) 191 279–281: 10.1016/j.tvjl.2011.06.044. 10.1016/j.tvjl.2011.06.044 22005167

[29] Roepman P, Wessels LF, Kettelarij N, Kemmeren P, Miles AJ, et al (2005) An expression profile for diagnosis of lymph node metastases from primary head and neck squamous cell carcinomas. Nature Genetics 37 182–186: 10.1038/ng1502. 10.1038/ng1502 15640797

[30] van de Peppel J, Kemmeren P, van Bakel H, Radonjic M, van Leenen D, et al (2003) Monitoring global messenger RNA changes in externally controlled microarray experiments. EMBO Reports 4 387–393: 10.1038/sj.embor.embor798. 10.1038/sj.embor.embor798 PMC1319154 12671682

[31] Yang YH, Dudoit S, Luu P, Lin DM, Peng V, et al (2002) Normalization for cDNA microarray data: A robust composite method addressing single and multiple slide systematic variation. Nucleic Acids Research 30: e15. 11842121 10.1093/nar/30.4.e15 PMC100354

[32] Margaritis T, Lijnzaad P, van Leenen D, Bouwmeester D, Kemmeren P, et al (2009) Adaptable gene-specific dye bias correction for two-channel DNA microarrays. Molecular Systems Biology 5 266: 10.1038/msb.2009.21. 10.1038/msb.2009.21 PMC2683724 19401678

[33] Wu H, Kerr MK, Cui X, Churchill GA (2003) MAANOVA: A software package for the analysis of spotted cDNA microarray experiments. In: Parmigiani GG, Garrett ES, Irizarri RA, Zeger SL, editors. The analysis of gene expression data: methods and software. New York: Springer. 313–339.

[34] Edgar R, Domrachev M, Lash AE (2002) Gene expression omnibus: NCBI gene expression and hybridization array data repository. Nucleic Acids Research 30: 207–210. 11752295 10.1093/nar/30.1.207 PMC99122

[35] Han JS, Szak ST, Boeke JD (2004) Transcriptional disruption by the L1 retrotransposon and implications for mammalian transcriptomes. Nature 429: 268–74. 15152245 10.1038/nature02536

[36] Adeagbo AS, Breen CA, Cutz E, Lees JG, Olley PM, et al (1990) Lamb ductus venosus: Evidence of a cytochrome P-450 mechanism in its contractile tension. J Pharmacol Exp Ther 252: 875–9. 2313604

[37] Nukaya M, Moran S, Bradfield CA (2009) The role of the dioxin-responsive element cluster between the Cyp1a1 and Cyp1a2 loci in aryl hydrocarbon receptor biology. Proc Natl Acad Sci U S A. 10.1073/pnas.0809613106 PMC2651201 19261855

[38] Court MH (1999) Anesthesia of the sighthound. Clinical Techniques in Small Animal Practice 14 38–43: 10.1016/S1096–2867(99)80025-5. 10.1016/S1096-2867(99)80025-5 10193044

[39] Walisser JA, Bunger MK, Glover E, Harstad EB, Bradfield CA (2004) Patent ductus venosus and dioxin resistance in mice harboring a hypomorphic arnt allele. J Biol Chem 279: 16326–31. 14764592 10.1074/jbc.M400784200

[40] Maltepe E, Keith B, Arsham AM, Brorson JR, Simon MC (2000) The role of ARNT2 in tumor angiogenesis and the neural response to hypoxia. Biochemical and Biophysical Research Communications 273: 231 – 238 10.1006/bbrc.2000.2928 10873592 10.1006/bbrc.2000.2928

[41] Wondimu A, Weir L, Robertson D, Mezentsev A, Kalachikov S, et al. (2012) Loss of arnt (Hif1beta) in mouse epidermis triggers dermal angiogenesis, blood vessel dilation and clotting defects. Laboratory Investigation; a Journal of Technical Methods and Pathology 92: 110–124. 10.1038/labinvest.2011.134 10.1038/labinvest.2011.134 21946855

[42] Sekine H, Mimura J, Yamamoto M, Fujii-Kuriyama Y (2006) Unique and overlapping transcriptional roles of arylhydrocarbon receptor nuclear translocator (arnt) and Arnt2 in xenobiotic and hypoxic responses. The Journal of Biological Chemistry 281 37507–37516: 10.1074/jbc.M606910200 17023418 10.1074/jbc.M606910200

[43] Kazlauskas A, Sundstrom S, Poellinger L, Pongratz I (2001) The hsp90 chaperone complex regulates intracellular localization of the dioxin receptor. Molecular and Cellular Biology 21: 2594 –2607 10.1128/MCB.21.7.2594.2607.2001 11259606 10.1128/MCB.21.7.2594-2607.2001 PMC86890

[44] Katschinski DM, Le L, Schindler SG, Thomas T, Voss AK, et al (2004) Interaction of the PAS B domain with HSP90 accelerates hypoxia-inducible factor-1alpha stabilization. Cellular Physiology and Biochemistry : International Journal of Experimental Cellular Physiology, Biochemistry, and Pharmacology 14 14:351–360 10.1159/000080345 10.1159/000080345 15319539

