# Supplementary material for: Altered Subcellular Localization of Heat Shock Protein 90 Is Associated with Impaired Expression of the Aryl Hydrocarbon Receptor Pathway in Dogs
Source: PLoS One. 2013 Mar 5;8(3):e57973. doi: 10.1371/journal.pone.0057973 (PMC3589449; doi:10.1371/journal.pone.0057973)
Supplement: Table S1 — primer sequences and annealing temperatures genomic DNA PCR. (DOCX) [file pone.0057973.s002.docx]

| **Gene** | **EnsemblID** | **exon** | **orientation** | **primersequence** | **application** |
| --- | --- | --- | --- | --- | --- |
| *AHR* | ENSCAFG00000002448 | 1 | f | GCCGATGTAGAAGGCACCTG | gPCR |
| *AHR* | ENSCAFG00000002448 | 1 | r | GAGCTCTGGACGGCGAAGAT | gPCR |
| *AHR* | ENSCAFG00000002448 | 1 | f | ATCACCTACGCCAGCCGCAA | RTPCR |
| *AHR* | ENSCAFG00000002448 | 2 | f | ACCCACTCCCTTGTTTTTCT | gPCR |
| *AHR* | ENSCAFG00000002448 | 2 | r | TCATTTAAGTGGCACCATCT | gPCR |
| *AHR* | ENSCAFG00000002448 | 2 | r | ATTGGAAGGAGAAGTGGAAC | gPCR / LINE-detection |
| *AHR* | ENSCAFG00000002448 | 2 | r | GACTTGATTCCTTCAGCTGG | RTPCR |
| *AHR* | ENSCAFG00000002448 | 2 | f | CCAGCTGAAGGAATCAAGTC | RTPCR / LINE-detection |
| *AHR* | ENSCAFG00000002448 | 3 | f | AGCCCTTTGAAGTCACTTAC | gPCR |
| *AHR* | ENSCAFG00000002448 | 3 | r | TCACATACTAGCGACACAAC | gPCR |
| *AHR* | ENSCAFG00000002448 | 3 | r | TTGCAGATGCAGACCTTCTC | RTPCR |
| *AHR* | ENSCAFG00000002448 | 4 | f | TGATAACCGTAACTGAAGTC | gPCR |
| *AHR* | ENSCAFG00000002448 | 4 | r | CCCTAACCTGAATGTTTCTT | gPCR |
| *AHR* | ENSCAFG00000002448 | 5 | f | CAAGCACTCAGTAATCTAGC | gPCR |
| *AHR* | ENSCAFG00000002448 | 5 | r | TATACATCTCCCAGAGGTTC | gPCR |
| *AHR* | ENSCAFG00000002448 | 5 | r | TCACCTCGGTCTTCAGTATG | RTPCR |
| *AHR* | ENSCAFG00000002448 | 6 | f | GTATCAGAACAGAATCCAAT | gPCR |
| *AHR* | ENSCAFG00000002448 | 6 | r | CCAAAGAAAGTAGTTTCAGT | gPCR |
| *AHR* | ENSCAFG00000002448 | 6 | f | GGTGTCTGCTGGATAATTCG | RTPCR |
| *AHR* | ENSCAFG00000002448 | 7 | f | GGAATTATCAAGACTACTGG | gPCR |
| *AHR* | ENSCAFG00000002448 | 7 | r | GCTAAGAGATTTACTGCAAC | gPCR |
| *AHR* | ENSCAFG00000002448 | 8 | f | TCAAGGAAGACACAATGCAG | gPCR |
| *AHR* | ENSCAFG00000002448 | 8 | r | TGGCAGCTATAGAGCACTTT | gPCR |
| *AHR* | ENSCAFG00000002448 | 9 | f | GTTAGAGGGGCTTTGTTTCA | gPCR |
| *AHR* | ENSCAFG00000002448 | 9 | r | AAACCCTGGAGTTTTCATTG | gPCR |
| *AHR* | ENSCAFG00000002448 | 9 | r | TCATGCCACTCTCTCCTGTC | RTPCR |
| *AHR* | ENSCAFG00000002448 | 10.1 | f | CTCCCTGCTCTCAAACTCAA | gPCR |
| *AHR* | ENSCAFG00000002448 | 10.1 | r | GGGTGTGATATTGTCTTGCC | gPCR |
| *AHR* | ENSCAFG00000002448 | 10.2 | f | CCATGATGCAACAAGATGAG | gPCR |
| *AHR* | ENSCAFG00000002448 | 10.2 | r | GCTATGTGATTTTGGTGGTG | gPCR |
| *AHR* | ENSCAFG00000002448 | 10.3 | f | CTCTGAACTCCAGCTGTATG | gPCR |
| *AHR* | ENSCAFG00000002448 | 10.3 | r | GGAAAGTCTAACTGTGTCCC | gPCR |
| *AHR* | ENSCAFG00000002448 | 10.4 | f | ATGCTATGCCATGTACACAG | gPCR |
| *AHR* | ENSCAFG00000002448 | 10.4 | r | AGAAGGATCACTGAAAGGGT | gPCR |
| *AHR* | ENSCAFG00000002448 | 11 | f | GTGCTCAGTAGGTCTTTGCT | gPCR |
| *AHR* | ENSCAFG00000002448 | 11 | r | TGGTGATGACCAACACTGAC | gPCR |
| *AIP* | ENSCAFG00000011515 | marker 1 | f | CTGATTCGTCCTACCCATCCTG | microsatellite |
| *AIP* | ENSCAFG00000011515 | marker 1 | r | GATTGTTTACATCCCGTCTTCCCT | microsatellite |
| *AIP* | ENSCAFG00000011515 | marker 2 | f | GACATGGGACTGTGATCCTG | microsatellite |
| *AIP* | ENSCAFG00000011515 | marker 2 | r | CTTGAGACTAAGAAGTTTCAGGG | microsatellite |
| *ARNT* | ENSCAFG00000012149 | marker 1 | f | ATGGGCCCAATCTCACAGTT | microsatellite |
| *ARNT* | ENSCAFG00000012149 | marker 1 | r | CTGACTCCAAGGGGGTTCTG | microsatellite |
| *ARNT* | ENSCAFG00000012149 | marker 2 | f | ACCCTTTGCCTGTGCTTTGT | microsatellite |
| *ARNT* | ENSCAFG00000012149 | marker 2 | r | TAAACAGGTGCCCAATGCAG | microsatellite |
| *CYP1A1/CYP1A2* | ENSCAFG00000017937 / ENSCAFG00000017941 | marker 1 | f | AAGATGGCTCTGGCTCTAGTG | microsatellite |
| *CYP1A1/CYP1A2* | ENSCAFG00000017937 / ENSCAFG00000017941 | marker 1 | r | TCAGATGCAAACTATTGAAGGG | microsatellite |
| *CYP1A1/CYP1A2* | ENSCAFG00000017937 / ENSCAFG00000017941 | marker 2 | f | GAATCCTCATCCCTTCTCCA | microsatellite |
| *CYP1A1/CYP1A2* | ENSCAFG00000017937 / ENSCAFG00000017941 | marker 2 | r | CAGTCATTTCCTGAACTCTCC | microsatellite |
| *CYP1B1* | ENSCAFG00000006164 | 2 | f | CAACGTCATGAGCGCCGTGT | gPCR |
| *CYP1B1* | ENSCAFG00000006164 | 2 | r | AGACTCTGGGCGTGCGTGGA | gPCR |
| *CYP1B1* | ENSCAFG00000006164 | 3 | f | GTGCCATGTGCTTTCTAGAT | gPCR |
| *CYP1B1* | ENSCAFG00000006164 | 3 | r | GAGGCTAATTGAGAGAGTGG | gPCR |
| *CYP1B1* | ENSCAFG00000006164 | 1&2 | f | GCTCCTTTCCCTGCACACCT | gPCR |
| *CYP1B1* | ENSCAFG00000006164 | 1&2 | r | CACACGGCGCTCATGACGTT | gPCR |
| *CYP1B1* | ENSCAFG00000006164 | marker 1 | f | ATAGAATAGCCCTTTCTTTCGGAG | microsatellite |
| *CYP1B1* | ENSCAFG00000006164 | marker 1 | r | ACTTTAGGGTAGGTAGGTGGG | microsatellite |
| *CYP1B1* | ENSCAFG00000006164 | marker 2 | f | CTCTTGGTTTCAGCTCAGGTC | microsatellite |
| *CYP1B1* | ENSCAFG00000006164 | marker 2 | r | AGTGTCAGCAGTATTCTTAGGA | microsatellite |
| *EDN1* | ENSCAFG00000009794 | marker 1 | f | TTAGATGTAGACTCACCTTTCTCC | microsatellite |
| *EDN1* | ENSCAFG00000009794 | marker 1 | r | AGACCGCATAATACCTCACAC | microsatellite |
| *EDN1* | ENSCAFG00000009794 | marker 2 | f | GATGTAGCCCAGACTATTCCA | microsatellite |
| *EDN1* | ENSCAFG00000009794 | marker 2 | r | TTTATCCCAGTGACCCAAACC | microsatellite |
| *HIF1A* | ENSCAFG00000015718 | 5 | f | CACCTTTGTGCTACTCTGTG | gPCR |
| *HIF1A* | ENSCAFG00000015718 | 5 | r | TCCCTCACAATTCTAATGCT | gPCR |
| *HIF1A* | ENSCAFG00000015718 | 6 | f | TGCCTGAGTTACATGAGTGG | gPCR |
| *HIF1A* | ENSCAFG00000015718 | 6 | r | GAAGGAAGCATTCGCAGTCT | gPCR |
| *HIF1A* | ENSCAFG00000015718 | 7 | f | TCAGTTACTTTGGAGAAGCA | gPCR |
| *HIF1A* | ENSCAFG00000015718 | 7 | r | CTCAGGTCTTGATCTCAGGT | gPCR |
| *HIF1A* | ENSCAFG00000015718 | 8 | f | CTCTGCTTGACTCTTTCTCC | gPCR |
| *HIF1A* | ENSCAFG00000015718 | 8 | r | GATGCGAATAGATGGCTACT | gPCR |
| *HIF1A* | ENSCAFG00000015718 | 9 | f | CCCACCCTATTGTGTCAATG | gPCR |
| *HIF1A* | ENSCAFG00000015718 | 9 | r | GGCTTGCAGCAACAGATAGA | gPCR |
| *HIF1A* | ENSCAFG00000015718 | 10 | f | GGACCTGTCAACAGTAGATT | gPCR |
| *HIF1A* | ENSCAFG00000015718 | 10 | r | GTTCTCTGTGGATGTCTAAA | gPCR |
| *HIF1A* | ENSCAFG00000015718 | 13 | f | TATTCCTTAATGTTGGCAAA | gPCR |
| *HIF1A* | ENSCAFG00000015718 | 13 | r | GCAGACACGTAACTGACTGA | gPCR |
| *HIF1A* | ENSCAFG00000015718 | 14 | f | TTTAGGGCCGTATTTAAGAA | gPCR |
| *HIF1A* | ENSCAFG00000015718 | 14 | r | AGCTATTCCTGCGTTACGAC | gPCR |
| *HIF1A* | ENSCAFG00000015718 | 15 | f | AGCTTATTTGATTTTCCTGC | gPCR |
| *HIF1A* | ENSCAFG00000015718 | 15 | r | ATGTGCTGTCTGTGATCTGG | gPCR |
| *HIF1A* | ENSCAFG00000015718 | 1&2 | f | GGACCTCTTGTGAGAATTCC | gPCR |
| *HIF1A* | ENSCAFG00000015718 | 1&2 | r | GGAGTGAGAGTATGACCACC | gPCR |
| *HIF1A* | ENSCAFG00000015718 | 11&12 | f | GTGTATTCTCTGAGGTCTGG | gPCR |
| *HIF1A* | ENSCAFG00000015718 | 11&12 | r | GGTGATGTGGTTATTTTCAA | gPCR |
| *HIF1A* | ENSCAFG00000015718 | 3&4 | f | GTGGTTTGCTTGCTATGTTA | gPCR |
| *HIF1A* | ENSCAFG00000015718 | 3&4 | r | GCAGATTTCACAAAGAACAG | gPCR |
| *HIF1A* | ENSCAFG00000015718 | marker 1 | f | TGGCCTCTCAGTGAAGTGAA | microsatellite |
| *HIF1A* | ENSCAFG00000015718 | marker 1 | r | TCCTGCAGACGAGGCATAGA | microsatellite |
| *HIF1A* | ENSCAFG00000015718 | marker 2 | f | ACCGCTGCCAAGAAGATGAT | microsatellite |
| *HIF1A* | ENSCAFG00000015718 | marker 2 | r | CCCTGCCATTTGATTGCTTT | microsatellite |
| *HSP90AA1* | ENSCAFG00000018036 | 11&12 | f | GTACTTGGAGTCCTTAGGTT | gPCR |
| *HSP90AA1* | ENSCAFG00000018036 | 11&12 | r | GTCATGCCTTACAGATTCTT | gPCR |
| *HSP90AA1* | ENSCAFG00000018036 | 2&3 | f | ATAACCTGGGTACCATCGCC | gPCR |
| *HSP90AA1* | ENSCAFG00000018036 | 2&3 | r | ACACCCCATGTGGACACTCA | gPCR |
| *HSP90AA1* | ENSCAFG00000018036 | 4&5 | f | TTCAGAGCGTGTTCAACAGC | gPCR |
| *HSP90AA1* | ENSCAFG00000018036 | 4&5 | r | CAGCACTTTCAGGAACTGGA | gPCR |
| *HSP90AA1* | ENSCAFG00000018036 | 6&7&8 | f | CTGGAAGTTCTGGGACAGAG | gPCR |
| *HSP90AA1* | ENSCAFG00000018036 | 6&7&8 | r | AGGAAGTTGGAAGAACAAGC | gPCR |
| *HSP90AA1* | ENSCAFG00000018036 | 9&10 | f | CTTGTGTGGTTAGGTCTGGC | gPCR |
| *HSP90AA1* | ENSCAFG00000018036 | 9&10 | r | AGGCACACTGAAGCACTGTT | gPCR |
| *HSP90AA1* | ENSCAFG00000018036 | marker 1 | f | ATCTGCTTCATCCCTCTCCA | microsatellite |
| *HSP90AA1* | ENSCAFG00000018036 | marker 1 | r | AGCCACTTCCATTCAACACC | microsatellite |
| *HSP90AA1* | ENSCAFG00000018036 | marker 2 | f | CACTGATCAAAGGAAAGCCAGAG | microsatellite |
| *HSP90AA1* | ENSCAFG00000018036 | marker 2 | r | CTTAACCAATAAGCCAGCCAG | microsatellite |
| *M13* |  |  | f | GTTTTCCCAGTCACGAC 6-FAM | microsatellite |
| *NOS3* | ENSCAFG00000004687 | marker 1 | f | AATGAGTGCGTGATAGAGTGTG | microsatellite |
| *NOS3* | ENSCAFG00000004687 | marker 1 | r | ATTCTGCATCGTTCAGCTCCA | microsatellite |
| *NOS3* | ENSCAFG00000004687 | marker 2 | f | CAGGCAAGTTACAGGTTACAG | microsatellite |
| *NOS3* | ENSCAFG00000004687 | marker 2 | r | TACAACCCAGAAGATTCCAG | microsatellite |
| *VEGFA* | ENSCAFG00000001938 | marker 1 | f | ACCCCTCTTCATGCTCTCTG | microsatellite |
| *VEGFA* | ENSCAFG00000001938 | marker 1 | r | AGCATATCAGTAGGGAAGGGAG | microsatellite |
