# Supplementary material for: Altered Subcellular Localization of Heat Shock Protein 90 Is Associated with Impaired Expression of the Aryl Hydrocarbon Receptor Pathway in Dogs
Source: PLoS One. 2013 Mar 5;8(3):e57973. doi: 10.1371/journal.pone.0057973 (PMC3589449; doi:10.1371/journal.pone.0057973)
Supplement: Table S2 — primer sequences and annealing temperatures used for quantitative PCR. B2M = β-2-Microglobulin, GAPDH = Glyceraldehyde-3-phosphatedehydrogenase, GUSB = beta-glucuronidase precursor, HNRPH = Heterogeneous nuclear ribonucleoprotein H, HPRT = hypoxanthine-guanine phosphoribosyltransferase, RPS5 = Ribosomal protein S5, AHR = aryl hydrocarbon receptor, AIP = aryl hydrocarbon receptor interacting protein, ARNT = aryl hydrocarbon receptor nuclear translocator, CYP1A1 = cytochrome P450, family 1, subfamily A, polypeptide 1, CYP1A2 = cytochrome P450, family 1, subfamily A, polypeptide 2, CYP1B1 = cytochrome P450, family 1, subfamily B, polypeptide 1, EDN1 = Endothelin-1, HIF1A = Hypoxia-inducible factor 1 alpha, HSP90AA1 = heat shock protein 90kDa alpha (cytosolic), class A member 1, NOS3 = nitric oxide synthase, endothelial, VEGFA = Vascular endothelial growth factor α (DOCX) [file pone.0057973.s003.docx]

| **Gene** | **EnsemblGeneID** | **primer** | **sequence** | **temp** | **length (bp)** | **2-step/3-step** |
| --- | --- | --- | --- | --- | --- | --- |
| *B2M* | ENSCAFG00000013633 | f | TCCTCATCCTCCTCGCT | 61.2 | 85 | 2 |
|  |  | r | TTCTCTGCTGGGTGTCG |  |  |  |
| *GAPDH* | ENSCAFG00000015077 | f | TGTCCCCACCCCCAATGTATC | 58 | 100 | 2 |
|  |  | r | CTCCGATGCCTGCTTCACTACCTT |  |  |  |
| *GUSB* | ENSCAFG00000010193 | f | AGACGCTTCCAAGTACCCC | 62 | 103 | 2 |
|  |  | r | AGGTGTGGTGTAGAGGAGCAC |  |  |  |
| *hnRPH* | ENSCAFG00000000336 | f | CTCACTATGATCCACCACG | 61.2 | 151 | 2 |
|  |  | r | TAGCCTCCATAACCTCCAC |  |  |  |
| *HPRT* | ENSCAFG00000018870 | f | AGCTTGCTGGTGAAAAGGAC | 56 | 114 | 3 |
|  |  | r | TTATAGTCAAGGGCATATCC |  |  |  |
| *RPS5* | ENSCAFG00000002366 | f | TCACTGGTGAGAACCCCCT | 62.5 | 141 | 2 |
|  |  | r | CCTGATTCACACGGCGTAG |  |  |  |
| *AHR* | ENSCAFG00000002448 | f | TTAGGCTCAGTGTCAGTTACC | 61 | 81 | 2 |
|  |  | r | ACTTCATTTCTGTCAGTTGGG |  |  |  |
| *AIP* | ENSCAFG00000011515 | f | CCTCAGCCTCTCATCTTTGAC | 64 | 103 | 2 |
|  |  | r | ACTGCCTTTGCCTTCTCC |  |  |  |
| *ARNT* | ENSCAFG00000012149 | f | GAACGACGACGACGGAACAAGATG | 63 | 83 | 2 |
|  |  | r | TTTTCGAGCCAGGGCACTACAGGT |  |  |  |
| *ARNT2* | ENSCAFG00000013922 | f | GCTTCACCTTCCAGAATCCC | 62 | 130 | 2 |
|  |  | r | GTCATACGACGACAACCCA |  |  |  |
| *CYP1A1* | ENSCAFG00000017937 | f | GAAGCCCCTGAGCCCAATGACT | 64 | 163 | 2 |
|  |  | r | CTGGTGTAGCCTGCTCTGAATGTTT |  |  |  |
| *CYP1A2* | ENSCAFG00000017941 | f | CACCATCCCCCACAGCACAACAAA | 59.7 | 139 | 2 |
|  |  | r | GCTCTGGCCGGAATGCAAATGGAT |  |  |  |
| *CYP1B1* | ENSCAFG00000006164 | f | TTTCACCAGGTATCCACAAGTG | 56.5 | 140 | 3 |
|  |  | r | GAGAAACGCATGCCTTCG |  |  |  |
| *EDN1* | ENSCAFG00000009794 | f | TGTCTACTTCTGCCACCT | 62 | 177 | 2 |
|  |  | r | AGTCCAGCACTTCTTGTC |  |  |  |
| *HIF1A* | ENSCAFG00000015718 | f | TTACGTTCCTTCGATCAGTTGTCA | 61 | 105 | 2 |
|  |  | r | GAGGAGGTTCTTGCATTGGAGTC |  |  |  |
| *HSP90AA1* | ENSCAFG00000025029 | f | CTTGACCGATCCCAGTAAGC | 59 | 128 | 3 |
|  |  | r | TATTGATCAGGTCGGCCTTC |  |  |  |
| *NOS3* | ENSCAFG00000004687 | f | GGCATACAGGACACAGGA | 62 | 176 | 2 |
|  |  | r | GCAATACCCGTACCAGGA |  |  |  |
| *VEGFA* | ENSCAFG00000001938 | f | CTTTCTGCTCTCCTGGGTGC | 58 | 101 | 2 |
|  |  | r | GGTTTGTGCTCTCCTCCTGC |  |  |  |
