# Supplementary material for: Altered Subcellular Localization of Heat Shock Protein 90 Is Associated with Impaired Expression of the Aryl Hydrocarbon Receptor Pathway in Dogs
Source: PLoS One. 2013 Mar 5;8(3):e57973. doi: 10.1371/journal.pone.0057973 (PMC3589449; doi:10.1371/journal.pone.0057973)
Supplement: Table S3 — Samples from dogs with extrahepatic portosystemic shunts (EHPSS) or intrahepatic portosystemic shunts (IHPSS) used for microarray and qPCR. (DOCX) [file pone.0057973.s004.docx]

|  |  | **Microarray** | | **qPCR** | |
| --- | --- | --- | --- | --- | --- |
| **Breed** | **Shunttype** | **Female** | **Male** | **Female** | **Male** |
| Cairn terrier | EHPSS | 3 | 4 | 0 | 0 |
| Cross breed | EHPSS | 2 | 1 | 0 | 0 |
| Jack Russell terrier | EHPSS | 3 | 3 | 0 | 0 |
| Maltese terrier | EHPSS | 3 | 2 | 0 | 0 |
| Miniature dachshund | EHPSS | 1 | 0 | 0 | 0 |
| Norfolk terrier | EHPSS | 2 | 1 | 0 | 0 |
| Shih Tzu | EHPSS | 1 | 0 | 0 | 0 |
| West Highland white terrier | EHPSS | 2 | 0 | 0 | 0 |
| Yorkshire terrier | EHPSS | 4 | 0 | 0 | 0 |
| Australian shepherd | IHPSS | 1 | 0 | 1 | 0 |
| Bearded collie | IHPSS | 0 | 1 | 0 | 0 |
| Bernese mountain dog | IHPSS | 2 | 1 | 0 | 1 |
| Cane corso | IHPSS | 0 | 1 | 0 | 0 |
| Duck tolling retriever | IHPSS | 0 | 1 | 0 | 1 |
| Golden retriever | IHPSS | 2 | 1 | 1 | 2 |
| Hovawart | IHPSS | 0 | 1 | 0 | 0 |
| Labrador retriever | IHPSS | 0 | 1 | 0 | 0 |
| Newfoundland | IHPSS | 1 | 0 | 1 | 0 |
| Deerhound | IHPSS | 0 | 0 | 0 | 1 |
| Beagle | control | 1 | 1 | 5 | 3 |
