# Supplementary material for: Altered Subcellular Localization of Heat Shock Protein 90 Is Associated with Impaired Expression of the Aryl Hydrocarbon Receptor Pathway in Dogs
Source: PLoS One. 2013 Mar 5;8(3):e57973. doi: 10.1371/journal.pone.0057973 (PMC3589449; doi:10.1371/journal.pone.0057973)
Supplement: Table S4 — Antibodies used for immunohistochemistry. TE = Tris-Ethylenediaminetetraacetic acid, RT = room temperature, O/N = over night, PBS = Phosphate buffered saline, TBS = Tris buffered saline. (DOCX) [file pone.0057973.s005.docx]

| **antigen** | **type** | **supplier** | **antigen retrieval** | **dilution** | **incubation primary antibody** | **washing buffer** | **addition to washing buffer** |
| --- | --- | --- | --- | --- | --- | --- | --- |
| anti-AHR | Rabbit polyclonal | Abcam  ab84833 | 40 min Citrate pH 6 | 1:500 | 1 hr RT | PBS | Tween-20 |
| anti-ARNT | Mouse monoclonal | Abnova  MAB2370 | 40 min Citrate pH 6 | 1:150 | O/N 4^o^C | PBS | Triton X100 |
| anti-CYP1A1 | Rabbit polyclonal | Santa Cruz Biotechnology  sc-20772 | 40 min TE pH 8 | 1:250 | O/N 4^o^C | PBS | Tween-20 |
| anti-CYP1B1 | Rabbit polyclonal | Abcam  ab78044 | 40 min TE pH 8 | 1:900 | O/N 4^o^C | PBS | Tween-20 |
| anti-CYP1A2 | Rabbit polyclonal | Abcam  ab77795 | 10 min protK (Dako) | 1:600 | O/N 4^o^C | PBS | Tween-20 |
| anti-HIF1A | Mouse monoclonal | Novus Biologicals  NB100-123 | 40 min TE pH 8 | 1:250 | O/N 4^o^C | PBS | Triton X100 |
| anti-HSP90AA1 | Mouse monoclonal | Novacastra  NCL-HSP90 | 30 min Citrate pH 6 | 1:10 | 1 hr RT | TBS | Tween-20 |
| anti-NOS3 | Rabbit polyclonal | Abcam  ab5589 | 40 min Citrate pH 6 | 1:100 | O/N 4^o^C | PBS | Tween-20 |
| anti-VEGFA | Rabbit polyclonal | Santa Cruz Biotechnology  sc-152 | 40 min TE pH 8 | 1:500 | O/N 4^o^C | PBS | Tween-20 |
