# Supplementary material for: Altered Subcellular Localization of Heat Shock Protein 90 Is Associated with Impaired Expression of the Aryl Hydrocarbon Receptor Pathway in Dogs
Source: PLoS One. 2013 Mar 5;8(3):e57973. doi: 10.1371/journal.pone.0057973 (PMC3589449; doi:10.1371/journal.pone.0057973)
Supplement: Table S5 — LOD scores for linkage of shunt phenotype with candidate genes from the AHR pathway in a digenic model. Genotypes of polymorphic microsatellites located close to candidate genes were analyzed assuming no recombinations occurred between markers and genes. (DOCX) [file pone.0057973.s006.docx]

|  | AHRR_m1 | AHRR_m2 | AIP_m1 | ARNT_m1 | ARNT_m2 | cyp1a1/2_m1 | cyp1a1/2_m2 | cyp1b1_m1 | cyp1b1_m2 | EDN1_m1 | Hif1A_m1 | Hif1A_m2 | HSP90AA1_m1 | HSP90AA1_m2 | NOS3_m1 | NOS3_m2 |
| --- | --- | --- | --- | --- | --- | --- | --- | --- | --- | --- | --- | --- | --- | --- | --- | --- |
| AHRR_m1 | LINKED | -0.55 | 0.13 | -0.10 | -0.73 | 0.51 | 0.91 | 0.33 | 0.22 | -0.19 | -0.60 | 0.10 | 0.68 | 0.52 | -0.11 | -0.10 |
| AHRR_m2 |  | LINKED | -0.01 | -0.21 | -0.87 | 0.39 | 0.76 | 0.22 | -0.02 | -0.26 | -0.75 | -0.01 | 0.58 | 0.39 | -0.21 | -0.21 |
| AIP_m1 |  |  | LINKED | 0.24 | -0.21 | 0.81 | **1.19** | 0.68 | 0.58 | 0.34 | -0.44 | 0.47 | 0.98 | 0.88 | 0.24 | 0.24 |
| ARNT_m1 |  |  |  | LINKED | -0.54 | 0.58 | 0.94 | 0.44 | 0.26 | 0.07 | -0.74 | 0.21 | 0.73 | 0.64 | 0.03 | 0.00 |
| ARNT_m2 |  |  |  |  | LINKED | 0.06 | 0.46 | -0.11 | 0.12 | -0.58 | -0.90 | -0.35 | 0.22 | 0.08 | -0.51 | -0.54 |
| cyp1a1/2_m1 |  |  |  |  |  | LINKED | **1.49** | **1.00** | 0.75 | 0.66 | -0.22 | 0.81 | **1.27** | **1.19** | 0.55 | 0.58 |
| cyp1a1/2_m2 |  |  |  |  |  |  | LINKED | **1.37** | **1.10** | **1.07** | 0.14 | **1.20** | **1.66** | **1.54** | 0.92 | 0.94 |
| cyp1b1_m1 |  |  |  |  |  |  |  | LINKED | 0.61 | 0.51 | -0.34 | 0.64 | **1.14** | **1.06** | 0.42 | 0.44 |
| cyp1b1_m2 |  |  |  |  |  |  |  |  | LINKED | 0.32 | -0.20 | 0.43 | 0.86 | 0.80 | 0.13 | 0.26 |
| EDN1_m1 |  |  |  |  |  |  |  |  |  | LINKED | -0.71 | 0.27 | 0.81 | 0.71 | 0.11 | 0.07 |
| Hif1A_m1 |  |  |  |  |  |  |  |  |  |  | LINKED | -0.77 | -0.08 | -0.17 | -0.80 | -0.74 |
| Hif1A_m2 |  |  |  |  |  |  |  |  |  |  |  | LINKED | 0.93 | 0.86 | 0.21 | 0.21 |
| HSP90AA1_m1 |  |  |  |  |  |  |  |  |  |  |  |  | LINKED | **1.33** | 0.67 | 0.73 |
| HSP90AA1_m2 |  |  |  |  |  |  |  |  |  |  |  |  |  | LINKED | 0.61 | 0.64 |
| NOS3_m1 |  |  |  |  |  |  |  |  |  |  |  |  |  |  | LINKED | 0.03 |
| NOS3_m2 |  |  |  |  |  |  |  |  |  |  |  |  |  |  |  | LINKED |
