# Supplementary material for: Altered Subcellular Localization of Heat Shock Protein 90 Is Associated with Impaired Expression of the Aryl Hydrocarbon Receptor Pathway in Dogs
Source: PLoS One. 2013 Mar 5;8(3):e57973. doi: 10.1371/journal.pone.0057973 (PMC3589449; doi:10.1371/journal.pone.0057973)
Supplement: Table S6 — Comparison of mRNA expression resulting from the microarray of genes of the AHR pathway. Relative mRNA expression of genes of the AHR pathway detected in microarray. Iw = Irish wolfhound, IHPSS = intrahepatic portosystemic shunt, EHPSS = extrahepatic portosystemic shunt. (DOCX) [file pone.0057973.s007.docx]

|  |  | *Microarray* | | | |
| --- | --- | --- | --- | --- | --- |
| **Gene Name** | **Ensembl Gene ID** | **IHPSS vs healthy** | **p-value** | **EHPSS vs healthy** | **p-value** |
| *AHR* | ENSCAFG00000002448 | 0.83 | 0.80 | 0.86 | 0.77 |
| *AIP* | ENSCAFG00000011515 | 1.00 | 1 | 0.96 | 1 |
| *ARNT* | ENSCAFG00000012149 | 1.04 | 1 | 0.99 | 1 |
| *ARNT2* | ENSCAFG00000013922 | 1.02 | 1 | 0.95 | 1 |
| *CYP1A1* | ENSCAFG00000017937 | 0.65 | 0 | 0.74 | 0 |
| *CYP1A2* | ENSCAFG00000017941 | 0.22 | 0 | 0.30 | 0 |
| *CYP1B1* | ENSCAFG00000006164 | 1.09 | 1 | 1.13 | 0.02 |
| *EDN1* | ENSCAFG00000009794 | 1.02 | 1 | 0.89 | 0.02 |
| *HIF1A* | ENSCAFG00000015718 | 1.36 | 0 | 1.13 | 1 |
| *HSP90AA1* | ENSCAFG00000018036 | 0.90 | 1 | 0.89 | 1 |
| *NOS3* | ENSCAFG00000004687 | 1.04 | 1 | 1.04 | 1 |
| *VEGFA* | ENSCAFG00000001938 | 1.02 | 1 | 0.97 | 1 |
